# The blood count as a compass to navigate in the ever-changing landscape of the carrier state of hemoglobinopathies: a single-center Italian experience

**DOI:** 10.3389/fped.2023.1228443

**Published:** 2023-10-06

**Authors:** Silvio Marchesani, Margherita Di Mauro, Giulia Ceglie, Ginevra Grassia, Michaela Carletti, Rosa Carmela Cristofaro, Matilde Cossutta, Cristina Curcio, Giuseppe Palumbo

**Affiliations:** ^1^ University Department of Pediatrics, Bambino Gesù Children’s Hospital, University of Rome Tor Vergata, Rome, Italy; ^2^ Department of Pediatric Hematology and Oncology, Cell and Gene Therapy, Bambino Gesù Children’s Hospital, IRCCS, Rome, Italy; ^3^ Department of Systems Medicine, University of Rome Tor Vergata, Rome, Italy; ^4^ Clinical Laboratory Unit, Bambino Gesù Children’s Hospital, IRCCS, Rome, Italy; ^5^ Medical Genetics Laboratory, Fondazione IRCCS Ca’ Granda Ospedale Maggiore Policlinico, Milan, Italy

**Keywords:** hemoglobinopathies, anemia, genetic testing, microcythemia, HPLC alterations

## Abstract

**Introduction:**

Approximately 7% of the worldwide population exhibits variations in the globin genes. The recent migration of populations from countries where hemoglobin disorders are endemic has resulted in important epidemiological changes with the diffusion of newly discovered or poorly characterized genetic variants and new combinations and very heterogeneous clinical phenotypes. The aim of our study is to assess the parameters that are more significant in predicting a positive genetic testing outcome for hemoglobinopathies in a pediatric population of patients presenting with anemia or microcythemia, without a definite diagnosis.

**Methods and materials:**

This study included patients evaluated in our hematological outpatient clinic for anemia and/or microcythemia despite normal ferritin levels. A screening of pathological hemoglobins using high-performance liquid chromatography (HPLC) was performed for the entire population of the study. Subsequently, patients with hemoglobin (Hb) S trait and patients with an HPLC profile compatible with beta thalassemia trait were excluded from the study. Genetic screening tests for hemoglobinopathies were performed on the remaining patients, which involved measuring the red blood cell (RBC) counts, red blood cells distribution width (RDW), reticulocyte count, and mean corpuscular volume of reticulocytes (MCVr).

**Results:**

This study evaluated a total of 65 patients, consisting of nine patients with negative genetic analysis results and 56 patients with positive genetic analysis results. The Hb and RDW values in these two groups did not demonstrate statistical significance. On the other hand, there were statistically significant differences observed in the mean corpuscular volume (MCV), RBC count, reticulocyte count, and MCVr between the two groups. Furthermore, in the group of patients with positive genetic test results, specific genetic findings associated with different HPLC results were observed. In particular, 13 patients with positive genetic test results had normal HPLC findings.

**Discussion:**

This study has demonstrated that HPLC, while serving as a valuable first-level test, has some limitations. Specifically, it has been observed that some patients may exhibit a negative HPLC result despite a positive genetic analysis. In addition to the presence of low levels of Hb and HPLC alterations, other parameters could potentially indicate the underlying mutations in the globin genes. Therefore, we propose that the complete blood cell count be utilized as a widely available parameter for conducting targeted genetic analyses to avoid the risk of overlooking rare hemoglobinopathies.

## 1. Introduction

Hemoglobinopathies, thalassemias, and hemoglobin variants, characterized by the abnormal structure of one or more globin chains, are the most common monogenic diseases (1).

Due to migratory flows in countries where thalassemia is endemic, there have been important epidemiological changes. In Italy, the genetic landscape of affected and carrier of hemoglobinopathies is in continuous evolution with the diffusion of many other hemoglobinopathies in addition to the beta thalassemia (2).

Hemoglobinopathies are very complex conditions for the genotypic aspect. In fact, more than 1,000 mutations responsible for abnormal hemoglobins have been described in the literature (3, 4), as well as for the phenotypic profile, considering that they are usually characterized by anemia of varying degrees, ranging from mild/moderate to a transfusion-dependent condition (5). The complete blood count, with particular attention to hemoglobin (Hb) levels, and the pathological hemoglobin screening [hemoglobin electrophoresis or high-performance liquid chromatography (HPLC)] represent the first approach to the study of these conditions (6).

HPLC is capable of detecting hemoglobin HbA, HbA2, HbF, and Hb variants in a single analysis. The results, when interpreted on the basis of the reference values, can identify individuals who carry or are affected by hemoglobinopathies. For example, HPLC is highly useful in identifying carriers of beta thalassemia due to the significant increase of HbA2 increase, which compensates for the poor synthesis of beta chains and is one of the most important markers of beta thalassemia carrier status (7). However, HPLC presents limitations to detect other thalassemic traits, such as alpha and delta thalassemia. In addition, HPLC has the capability to recognize the majority of the Hb variants, such as HbS, HbC, and Hb O’Arab and HbE in homozygosis. Overall, HPLC has the capacity to identify approximately 70% of the prevalent variants (8), but the screening shows limitations, resulting in the potential oversight of certain pathological hemoglobin variants.

Currently, the Hb level and HPLC alterations are suggestive of an underlying mutation in the globin genes, hence requiring further testing to assess the genetic etiology. However, some parameters of the complete blood count could also be useful in identifying these conditions.

The primary objective of this study is to determine whether specific **Complete Blood Cell count** parameters c**ould be used to predict a positive genetic testing for hemoglobinopathies in** a pediatric population presenting with anemia or microcythemia, without a definite diagnosis. By exploring the correlation between these hematological parameters and the presence of **genetically determined** hemoglobinopathies, this research aims to enhance the diagnostic accuracy and early detection of these conditions, thereby facilitating more effective clinical management.

## 2. Methods and materials

### 2.1. Population study

A retrospective and monocentric study was conducted on a pediatric population aged between 1 and 18 years. The time period under consideration was from 1 January 2020 to 31 May 2022. The data were obtained from the electronic medical records of the patients.

The patients included in this study were evaluated at our hematological outpatient clinic at the Bambino Gesù Children's Hospital for the presence of anemia and/or microcythemia, despite having normal ferritin levels (defined as below the age/specific limit of 2 standard deviations).

Other blood count indices were also considered in the initial assessment such as red blood cells (RBC) count, red blood cells distribution width (RDW), reticulocyte count, and mean corpuscular volume of reticulocytes (MCVr). For normal values, we considered the age/specific limit of 2 standard deviations.

ADVIA 2120i Hematology System was used for complete blood cell analysis.

Other information such as date of birth, age, sex, genotype, clinical and laboratory characteristics, and possible treatment were extracted from the electronical medical records.

The entire study population had a first-level diagnostic test in which HPLC was utilized to screen for pathological hemoglobins.

Subsequently, patients with HbS trait and patients with an isolated increase of HbA2 (HbA2 > 3.3%) suggestive for beta thalassemia trait were excluded from the study.

In order to assess the remaining patients who had normal or different anomalies in their HPLC results, a genetic test was conducted to screen for hemoglobinopathies. Prior to conducting the test, informed consent was obtained from the legal guardians of the patients.

**Figure 1:**
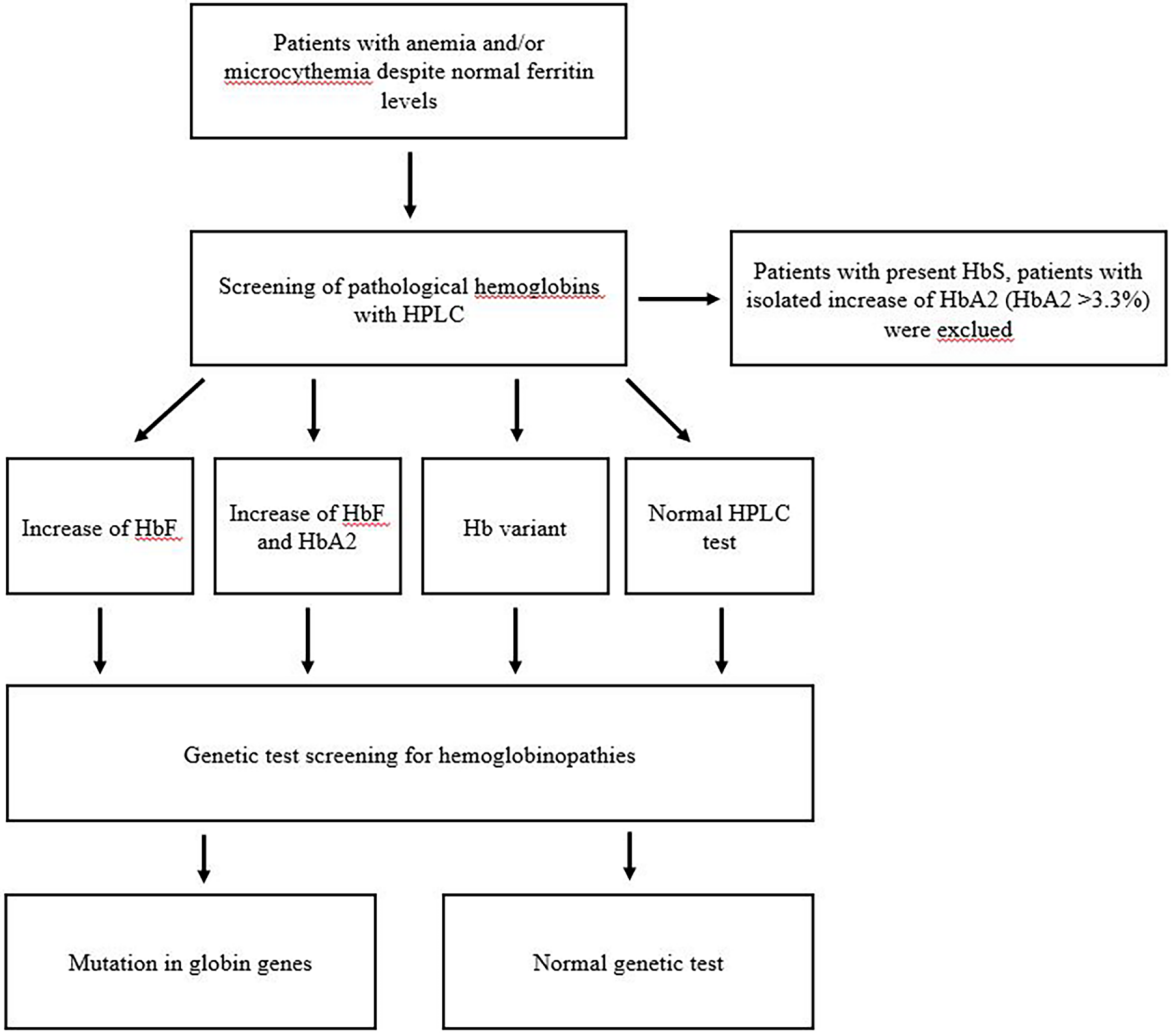
Management flowchart.

The management flowchart is shown in Figure 1.

### 2.2. High-pressure liquid chromatography

The separation and quantification of the hemoglobin fractions was performed using The VARIANT II (Bio-Rad), an HPLC instrument with ion exchange.

Our instrument is completely automatized working on a primary tube, able to obtain quantification and separation of the hemoglobin fractions in a few minutes from a single EDTA blood sample. The instrument has the ability of quantifying and recognizing the principal hemoglobin fractions (HbA, HBF, HbA2) and the hemoglobin variants (HbS, HbC, HbD) and to report abnormal peaks that can be identified as unknown and rare variants of the beta, alpha, and delta chain. HbE and Hb Lepore components are not separated and quantified since they elute with the HbA2 component.

HbF findings were considered normal for values between 0.1% and 1.2%. HbA2 findings were considered normal for values between 2% and 3.3%.

The HPLC result was evaluated as negative if HbF and HbA2 values were normal in the absence of hemoglobin variants.

### 2.3. Genetic analysis

Genomic DNA was extracted from peripheral blood leucocytes using an automatic nucleic acid extractor, QIASYMPHONY (QIAGEN). Molecular characterization of the globin gene mutations (alpha, beta, delta, gamma) was achieved by a polymerase chain reaction (PCR), followed by a direct DNA sequencing of the PCR products. Sequencing of the globin genes was performed by Sanger sequencing (BigDye Terminator Cycle Sequencing Ready Reaction Kit v.1.1) on the ABI Prism 3130 XL Genetic Analyzer (Applied Biosystems, Foster City, CA, USA).

A multiplex ligation–dependent probe amplification (MLPA) assay was employed using the SALSA MLPA Probemix P140-C1HBA (MRC-Holland, Amsterdam, Netherlands) and the SALSA MLPA Probemix P102-D1 HBB to detect deletions/duplications within the alpha gene and beta gene clusters, respectively.

We considered “positive genetic tests” the ones that diagnosed mutations in the globin genes.

### 2.4. Statistical analysis

A statistical analysis was performed to compare and correlate the data. Student’s *t*-test (two-sided) was used to compare two different groups for parametric distribution or Mann–Whitney test for non-parametric distribution. Chi-squared test or Fisher's exact test (when appropriate) was performed to compare proportions or categorical outcomes. Data considered with statistical significance were those with a *p*-value of less than 0.05. The statistical analysis was conducted using the GraphPad Prism software, version 5 for Macintosh (GraphPad Software, Inc.).

## 3. Results

### 3.1. Population study

In our center, a total of 68 patients presenting with anemia and/or microcythemia, despite having normal ferritin levels, were assessed between the period of 1 January 2020 and 31 June 2022. These patients exhibited normal or different anomalies in their HPLC results, which differed from the anomalies previously mentioned in the Methods and materials section. These patients underwent genetic testing.

Among those included in the study, nine patients exhibited a negative genetic analysis result, while 56 patients displayed a positive result. In the following sections, we summarized the clinical and laboratory findings of the different subpopulations.

### 3.2. Patients with a negative genetic testing

The genetic testing conducted on the nine patients (seven males and two females, aged 1–14 years old, median: 2 years old) did not reveal any alterations.

In this population, the median Hb level was found to be 9.3 g/dl (range 9–11.2 g/dl) at onset, while the median MCV value was 74.2 fl (range: 67–90 fl).

RBC count was on a median of 3,810,000/µl (range 36,100,000–5,030,000/µl), and the RDW median value was 17.2% (range: 14.7%–25.3%). The median reticulocyte count as absolute number was 61,000/µl (range: 10,500–135,000/µl), and the MCVr had a median value of 92.3 fl (88–109 fl).

Ferritin levels were normal in all patients as required by the inclusion criteria.

All patients underwent HPLC testing before the genetic assessment with the following results:

- HbF median value: 1.2% (range 0.2%–4.3%).
- HbA2 median value: 2.2% (range 1.1%–3.2%).

An isolated increase of HbF levels was detected in four patients, while the remaining five patients exhibited normal results.

### 3.3. Patients with a positive genetic testing

A total of 56 pediatric patients (33 males and 23 females) aged between 1 and 18 years (median age: 2 years) underwent genetic assessment that revealed anomalies in the globin genes.

In this population, the median Hb level was found to be 10.2 g/dl (range 7.6–14.4 g/dl) at onset, while the average MCV value was 63.5 fl (range: 45.2–91 fl). The median RBC count was 5,310,000 (range 2,860,000–6,520,000). The RDW median value was 17.5% (range 13%–26%). The reticulocyte count was on a median value of 98,000 (44,000–4,141,000/µl) while MCVr mean value was 84.8 (range 65–101 fl).

All patients underwent HPLC testing before the genetic assessment with the following results:

- HbF mean value: 3.2% (range 0.2%–83%).
- HbA2 mean value: 2.7% (range 0.1%–27%).

To better characterize the clinical and laboratory characteristics of this population, we further categorized it into four distinct groups based on their HPLC results (Figure 1). The four groups were as follows:

- Increase of HbF levels (19 patients, 33.9% of the total population, median age: 1 year, range: 1–12 years).
- Increase of HbF and HbA2 levels (18 patients, 32.1% of total population, median age: 4.5 years, range: 1–18 years).
- Presence of Hb variant, HbS excluded (six patients, 10.7% of the total population, median age: 5 years, range: 1–14 years).
- Normal HPLC test result (13 patients, 23.3% of the total population, median age: 4 years, range: 1–10 years).

The reticulocyte and MCVr values at onset were not available for nine patients, while the RBC count at onset was not available for three patients.

### 3.4. Statistical analysis

We then proceeded to compare the group of patients with a positive genetic testing and the group without alterations to investigate whether there are specific laboratory parameters that could predict a positive genetic outcome.

The Hb value did not demonstrate statistical significant with a *p*-value of 0.14. Similarly, the RDW value also lacked statistical significance with a *p*-value of 0.95. On the other hand, all the other parameters (MCV, RBC count, reticulocyte count in absolute number, MCVr) exhibited statistically significant differences between the two groups (see Table 1 for details).

**Table 1:** Comparison between patients with positive genetic tests (Genetic+) and patients with negative genetic tests (Genetic−).

Value	Genetic+ group	Genetic− group	*p*-value
Median age (range), years	2 (1–18)	2 (1–14)	0.98
Sex	Females 41% Males 59%	Females 22% Males 78%	0.28
Median Hb (range), g/dl	10.25 (7.6–14.4)	9.3 (9–11.2)	0.14
Median RBC (range), /µl	5,310,000 (2,860,000–6,520,000)	3,810,000 (3,610,000–5,030,000)	0.0004*
Median MCV (range), fl	63.5 (45.2–91)	74.2 (67.1–90.6)	0.003*
Median RDW (range), %	17.5 (13–26)	17.2 (14.7–25.3)	0.95
Median reticulocytes (range), /µl	98,000 (44,000–4,141,000)	61,000 (10,500–135,000)	0.011*
Median MCVr (range), fl	84.8 (65–101)	92.3 (87.8–108.7)	0.0008*

* Data with statistical significance.

Majority of the identified genotypes have only been anecdotally reported in the general population. Therefore, the general incidence in the Italian population cannot be retrieved and hence remains unreported in this context.

## 4. Discussion

In this study, we wanted to assess what parameters are more significant in predicting a positive genetic test for hemoglobinopathies in a pediatric population of patients with anemia.

Disorders of hemoglobin, due to genetic alterations in one or more of the globin genes, are the most common monogenic disorders worldwide. In fact, approximately 7% of the world population is heterozygous for one of these genes (1, 9). The diffusion of hemoglobinopathies worldwide is a constantly changing phenomenon, and the actual indications are set on an outdated historical context, in which the prevalence of globin variants was low and limited to some geographical areas. The recent migratory process of populations from countries where hemoglobin disorders are endemic has led to important epidemiological changes. Furthermore, we are witnessing an increased diffusion of new or little-known genetic variants and especially new combinations, which can be associated with very heterogeneous clinical phenotypes (2, 10).

To study the efficacy of genetics in diagnosing hemoglobinopathies, we selected a group of patients with anemia, or microcytic anemia, without a diagnosis. We excluded patients with low ferritin levels, as well as patients with iron deficiency anemia. Moreover, all patients were subjected to HPLC testing, and those diagnosed with beta thalassemia trait (HbA2 > 3.3%) or sickle cell trait (presence of HbS) were excluded. No HPLC or non-diagnostic alterations were observed in the remaining subjects.

Genetic testing and data analysis were performed in this group of patients.

The first relevant result found was the positive genetic test in the majority of the patients examined (56 out of 65 patients). Initially, the patients with positive genetic test were divided into two groups: (1) patients with abnormal HPLC results later confirmed through molecular analysis and (2) patients with no relevant findings during the HPLC testing, but were diagnosed exclusively based on genetic research studies.

The patients with HPLC alterations were categorized into three groups: those with an isolated increase of HbF levels, those with an increase of both HbF and HbA2 levels, and those with the presence of Hb variants.

A total of 19 patients with an isolated increase of HbF levels represent the first group. They exhibited heterogeneous clinical and genetic pictures. Overall, microcytic anemia was found to be the dominant clinical presentation. Some of these (four patients accounting for 22%) presented with severe anemia and required blood transfusions; specifically, the three patients diagnosed with beta thalassemia (beta0/beta0, beta0/beta + thalassemia or compound heterozygosis beta0/delta − beta0) required blood transfusions. However, despite the presence of underlying mutations, they showed a phenotype requiring infrequent blood transfusion support.

Although the HPLC alteration was the same, HbF levels increased. However, the genetic test showed different mutations. The increased production of HbF can occur through various means, including prematurity (11), bone marrow failure syndromes (12), leukemia (13, 14), solid tumors (15), or as an inherited disorder, the latter as a primary condition or as secondary to various anemias. The primary increases are divided in deletional and non-deletional forms. The deletional group is caused by deletions in the beta gene cluster, resulting in the removal of some parts or the entirety of the beta and delta-globin genes. These deletions are associated with a variable but often nearly complete compensatory increase in the expression of the gamma-globin gene and HbF levels. The non-deletional forms are caused by point mutations in the gamma-chain promoters. An increase in HbF levels is also observed in other cases of hemoglobinopathies such as beta thalassemia, delta beta thalassemia, and sickle cell anemia (16–18).

The increase in HbF and HbA2 levels represents the main alteration in the second category of patients, comprising a total of 18 patients. The majority of patients exhibited microcytic anemia. In addition, we found heterogeneous clinical and genetic pictures in this group. In fact, although the majority of cases have a benign clinical phenotype without complications, two patients (11.1%) required blood transfusions concomitantly with infectious events. Numerous mutations have been identified through genetic research. Moreover, genetic analysis played a crucial role in accurately diagnosing two hemoglobin variants that were not identified by HPLC. Specifically, one case involved the detection of HbE, while the other case involved the identification of Hb Lepore.

The last group of HPLC alterations pertains to the presence of Hb variants. Furthermore, different laboratory findings were documented in this group. Out of the six patients assessed, two patients displayed microcytic anemia, one patient had normocytic anemia, and three patients had microcythemia without anemia. None of the patients in this group received transfusion. HPLC identified the homozygous existence of Hb C, Hb O'Arab, and HbE.

Genetic analysis played a crucial role in identifying 20 different hemoglobin variants, out of which six cases (30%) initially went undetected using HLPC. In these cases, the genetic tests provided additional information that could not be determined otherwise. Specifically, the two patients who were heterozygous for HbC displayed a thalassemia trait known as alpha + (del − 3.7). Regarding the presence of HbE, three patients were identified through genetic testing. Among these patients, only one case was diagnosed through HPLC and confirmed to be homozygous for HbE through genetic analysis. However, HPLC testing was negative for the other two patients (who displayed heterozygosity for HbE). In addition, HPLC testing was negative for Hb Lepore, Hb Contaldo, Hb Sardinia, and Hb A2-NYU in these two patients.

Regarding the patients with normal HPLC results (13 out 56 patients), it was observed that they presented normal levels of HbA2 (<3.3%) and HbF (<1.2%), without any presence of Hb variants. However, these patients displayed alterations in their blood count, such as microcytic/normocytic anemia or microcythemia without anemia. Their genetic exams highlighted 10 carriers of alpha thalassemia and one carrier of delta thalassemia.

Alpha thalassemia is one of the most common monogenic gene disorders across the global population. The molecular basis typically involved deletions and, less frequently, point mutations affecting the expression of one or more of the duplicated alpha-genes. The clinical variation and increase in disease severity are directly related to the decreased expression of either one, two, three, or four copies of the alpha-globin genes (19). The two clinically significant forms of alpha thalassemia are hemoglobin Bart’s hydrops fetalis syndrome (caused by deletion/inactivation of all four alpha-globin genes; −/−) and hemoglobin H disease (caused by deletion/inactivation of three alpha-globin genes; −/− alpha) (20). The patients in our study exhibited the mild forms, specifically alpha + thalassemia (caused by deletion/inactivation of one alpha-globin genes; alpha-alpha/alpha−) and alpha 0 thalassemia (caused by deletion/inactivation of two alpha-globin genes: alpha-/alpha−, alpha-alpha/−). Alpha + thalassemia is marked solely by mild microcytosis; alpha0 thalassemia is characterized by mild microcytic anemia, a phenotypic expression very similar to the carrier of beta thalassemia (21). In both cases, hemoglobin electrophoresis is normal, and molecular studies are essential to identify mutations of the globin genes. Due to the high rate of carriers of alpha deletions, genetic screening is important to identify couples at risk of having a baby with Hb Bart syndrome or HbH disease (20).

In addition to patients with alpha thalassemia, genetic testing was able to diagnose a carrier of delta thalassemia among the group of patients with negative HPLC results. The patient exhibited a case of mild microcytic anemia. In fact, the mutations of the delta-globin gene solely decrease the quality or quantity of the delta-globin chain synthesis, without any clinical effect (22). Nevertheless, it is crucial to establish a diagnosis of delta thalassemia due to the potential impact of its interaction with defects in other globin genes, which can alter the percentage of Hb A2 and lead to an inaccurate diagnosis. For example, the elevated Hb A2 (alpha2delta2) levels typically indicate the presence of a beta thalassemia carrier; however, the coinheritance of delta and beta thalassemia may result in a normal Hb A2 level, as Hb A2 levels remain normal despite deficient delta chain production (23).

These results highlight the limitations of HPLC testing. Although it has the advantage of being easy to use and inexpensive, it showed many important limitations. The hemoglobin screening method successfully detected 70% of the diagnosed hemoglobin variants using genetic testing (8). In particular, it accurately identified the presence of Hb S, Hb C, Hb O’Arab, and HbE in homozygosis. However, the heterozygous HbE, Hb Lepore, Hb Contaldo, Hb Sardinia, and Hb A2-NYU were undetected. In addition, the screening process presented limitations in accurately identifying the alpha and delta thalassemia carriers, who had negative HPLC results but with pathological genetic panel.

To summarize, our findings confirm and reiterate the importance of genetic testing in the diagnosis of hemoglobinopathies, and considering the abundance of a heterogeneous group of different variants identified (alpha, beta, gamma, and delta), we emphasize the importance of studying all globins. Genetic testing is essential to make a diagnosis and therefore gain a better understanding of the clinical phenotype, therapeutic possibilities, and reproductive risk.

Given that HPLC has been observed to lack diagnostic accuracy in many patients who were later diagnosed through genetic testing, our study aimed to analyze our dataset to identify which blood count parameters may serve as predictors for a positive genetic test result. Hemoglobinopathies are usually characterized by varying degrees of anemia, ranging from mild or moderate conditions to transfusion-dependent diseases (5), extramedullary erythropoiesis, and early hemolysis of red blood cells (24, 25). In this study, our findings confirmed that the presence of anemia with normal ferritin values could lead the clinical suspect toward alterations in the globin genes. However, our findings did not demonstrate significantly lower anemia values in patients with positive genetic results (in patients with negative genetic results. the Hb mean value was of 9.3 vs. 10.2 g/dl for the patients with positive genetic results). The same was observed in RDW, where a quantitative measure of anisocytosis (mean value 17.2% for patients with negative genetics vs. 17.5% for patients with positive genetics) did not demonstrate statistical significance. On the other hand, other significant differences in blood count parameters were seen between patients with positive genetics and those with negative genetics (Table 1), including MCV, total number of red blood cells, reticulocytosis, and MCVr.

The average MCV value in the negative genetic testing population was found to be 92.3 fl, but in the positive genetic population, the average MCV value was seen to be 63.5 fl (*p*-value 0.03, Table 1). This finding indicates that low MCV level is not exclusively indicative of iron deficiency anemia or beta thalassemia carriers, but it is also a hallmark of other less common hemoglobinopathies or carrier status. Another difference between the two populations pertains to the total number of red blood cells (3,810,000/µl vs. 5,310,000/µl, Table 1). Lastly, there have been observed variations in the reticulocyte indices. The negative genetics sample had an absolute reticulocyte count of 61,000/µl and an MCVr mean value of 92.3 fl. In the positive genetics sample, the reticulocyte count was 98,000/µl, and the MCVr mean value was 84.8 fl (*p*-value 0.0008, Table 1). The RBC count and the reticulocyte indices are blood parameters extensively studied by previous research studies. In fact, the high RBC count and the mildly increased reticulocyte count are traditionally related to a beta thalassemia carrier, and they are often helpful in distinguishing thalassemia from iron deficiency anemia (26). Concerning MCVr, there is a limited number of studies available on its clinical utility. For example, MCVr was measured in subjects with depleted iron stores or megaloblastic anemia in order to monitor the efficacy of replacement therapy (27, 28).

Based on our analysis, red cell parameters and reticulocyte indices play an essential role in the differential diagnosis of anemia, and also provide a significant help in predicting a positive genetic testing for hemoglobinopathies.

Despite these interesting data, the laboratory alterations displayed an important heterogeneity, even among patients with identical genetic alterations. Consequently, the lack of a univocal correlation between genotype and phenotype implies a difficult interpretation of the results, presenting an ongoing challenge for physicians.

Although our study highlights some important issues regarding the diagnosis of hemoglobinopathies and the carrier status, it has some limitations. One critical point concerns patients with anemia/microcythemia but yielding negative results on genetic testing (nine out of 65 patients). In the group of nine patients with negative genetics, five patients had normal HPLC results. Specifically, four of these patients exhibited an isolated increase in HbF levels. Further investigation should be conducted to explore an additional genetic reason for the increased HbF observed in this subgroup of patients.

The persistence of HbF has emerged as a prominent issue of investigation. In recent years, understanding the regulation of fetal hemoglobin genes has been the subject of intensive study.

In general, HbF production is characteristic of the prenatal and perinatal period. In fact, during human erythroid development, there is a sequential switch from expression of the embryonic *ε*-globin gene to the fetal ɣ-globin gene *in utero*. Later on, in the postpartum period, the ɣ-globin gene is silenced, as the beta-globin gene becomes the predominantly expressed locus (29). The transcription regulatory complexes at the beta-globin gene govern this developmental switch. However, the silencing of the fetal globin genes in adult RBC count is incomplete, resulting in the limited amounts of HbF in the bloodstream of healthy adults. Furthermore, higher levels of HbF may be observed in patients with sickle cell disease and several types of beta thalassemia. The hereditary persistence of HbF (HPFH) is a condition resulting from a variety of different mutations. The major loci that account for the genetic variation in HbF are *BCL11A, KLF1, HBSL1-MYB* DNA region, *SOX6, FOP, NF-E4, TR2/TR4* erythroid-definitive complex, and *COUP-TFII* (30–33).

Most of these genes are involved in the gamma-chain downregulation, and in particular, the most studied is *BCL11A*, which was identified as a key gamma-globin gene repressor. These recent insights into the repressive factors that silence gamma-globin gene expression have offered potentially novel approaches to “reactivate” HbF expression for therapeutic purposes in the context of sickle cell disease and beta thalassemia (30). In fact, increasing the expression of HbF in adults is of major clinical interest in beta thalassemia, as it can potentially serve as a compensatory mechanism for the lack of HbA production. Similarly, in the cases of SCD, HbF provides a potent anti-sickling effect by blocking the deoxygenation-induced HbS polymerization (34).

Performing genetic tests aimed at identifying these regulatory genes could justify some of the patterns found in our population. Nevertheless, these genetic conditions have a benign course and protective effect toward other beta variants, so we do not routinely perform them in all patients with high HbF levels.

All current studies in Europe focus on the identification of the carriers of beta thalassemia during the preconception, pregnancy, or neonatal stages. In contrast, this study shows that the genetic landscape of these carriers is changing. In Italy, the most commonly found alteration is the presence of beta thalassemia carriers, even though the prevalence of other hemoglobinopathies is steadily increasing.

To the best of our knowledge, this is the first study that has extensively characterized patients with anemia or microcythemia to study parameters in the complete blood cell count that may indicate the necessity of genetic testing. These data confirm the importance of genetic testing in the diagnosis of hemoglobinopathies although it must be reserved to selected patients. We are aware that this study and its conclusions have several limitations. It is a retrospective study, with a small sample size and with an unequal distribution of participants across the two groups being compared (positive vs. negative genetic test sample). Moreover, it is a monocentric study, with the bias of the high incidence of hemoglobinopathies in Italy (35). Our study was conducted in cases of moderate anemia, especially microcytic, in which iron deficiency or other known causes have been excluded, with no HPLC alterations or non-diagnostic alterations. This study has demonstrated that HPLC, while serving as a valuable first-level test, has many limitations. Specifically, it has been observed that some patients may exhibit a negative HPLC result despite a positive genetic testing. Besides the presence of low levels of Hb and HPLC alterations, other parameters may be suggestive of underlying mutations in the globin genes, such as MCV, RBC count, MCVr, and reticulocytes count. Therefore, we propose utilizing these blood count parameters as a crucial tool in identifying patients who warrant complete genetic testing.

## Data Availability

The raw data supporting the conclusions of this article will be made available by the authors, without undue reservation.
